# Supplementary material for: Splice-Junction-Based Mapping of Alternative Isoforms in the Human Proteome
Source: Cell Rep. Author manuscript; Available in PMC 2020 Jan 15. (PMC6961840; doi:10.1016/j.celrep.2019.11.026)
Supplement: 2 [file NIHMS1546469-supplement-2.zip › DF1/PXD010154/Testis/Testis_29_SPTAN1_LPWETAEDIQER.pdf]

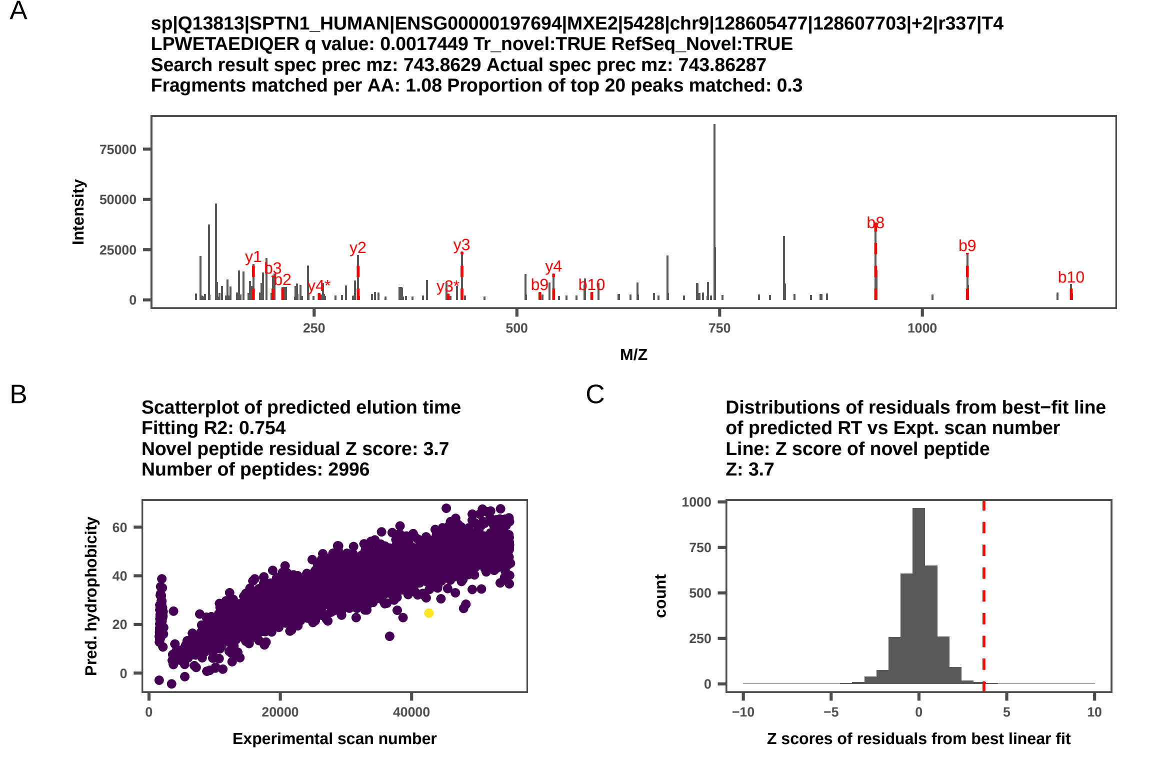
A
sp|Q13813|SPTN1_HUMAN|ENSG00000197694|MXE2|5428|chr9|128605477|128607703|+2|r337|T4
LPWETAEDIQER q value: 0.0017449 Tr_novel:TRUE RefSeq_Novel:TRUE
Search result spec prec mz: 743.8629 Actual spec prec mz: 743.86287
Fragments matched per AA: 1.08 Proportion of top 20 peaks matched: 0.3
Intensity
75000
50000
25000
0
y1
b3
b2
y4*
y2
y3
y3*
b9
y4
b10
b8
b9
b10
250
500
750
1000
M/Z
B
Scatterplot of predicted elution time
Fitting R2: 0.754
Novel peptide residual Z score: 3.7
Number of peptides: 2996
Pred. hydrophobicity
60
40
20
0
0
20000
40000
Experimental scan number
C
Distributions of residuals from best−fit line
of predicted RT vs Expt. scan number
Line: Z score of novel peptide
Z: 3.7
count
1000
750
500
250
0
−10
−5
0
5
10
Z scores of residuals from best linear fit
